# Clinically relevant genes and regulatory pathways associated with NRAS^Q61^ mutations in melanoma through an integrative genomics approach

**DOI:** 10.18632/oncotarget.2954

**Published:** 2014-12-10

**Authors:** Wei Jiang, Peilin Jia, Katherine E. Hutchinson, Douglas B. Johnson, Jeffrey A. Sosman, Zhongming Zhao

**Affiliations:** ^1^ Department of Biomedical Informatics, Vanderbilt University School of Medicine, Nashville, Tennessee, USA; ^2^ Center for Quantitative Sciences, Vanderbilt University School of Medicine, Nashville, Tennessee, USA; ^3^ Department of Cancer Biology, Vanderbilt University School of Medicine, Nashville, Tennessee, USA; ^4^ Vanderbilt-Ingram Cancer Center, Vanderbilt University School of Medicine, Nashville, Tennessee, USA; ^5^ Department of Medicine/Division of Hematology-Oncology, Vanderbilt University School of Medicine, Nashville, Tennessee, USA

**Keywords:** NRAS, melanoma, driver mutation, DNA methylation, gene expression, regulatory pathway

## Abstract

Therapies such as BRAF inhibitors have become standard treatment for melanoma patients whose tumors harbor activating BRAF^V600^ mutations. However, analogous therapies for inhibiting NRAS mutant signaling have not yet been well established. In this study, we performed an integrative analysis of DNA methylation, gene expression, and microRNA expression data to identify potential regulatory pathways associated with the most common driver mutations in NRAS (Q61K/L/R) through comparison of NRAS^Q61^-mutated melanomas with pan-negative melanomas. Surprisingly, we found dominant hypomethylation (98.03%) in NRAS^Q61^-mutated melanomas. We identified 1,150 and 49 differentially expressed genes and microRNAs, respectively. Integrated functional analyses of alterations in all three data types revealed important signaling pathways associated with NRAS^Q61^ mutations, such as the MAPK pathway, as well as other novel cellular processes, such as axon guidance. Further analysis of the relationship between DNA methylation and gene expression changes revealed 9 hypermethylated and down-regulated genes and 112 hypomethylated and up-regulated genes in NRAS^Q61^ melanomas. Finally, we identified 52 downstream regulatory cascades of three hypomethylated and up-regulated genes (*PDGFD, ZEB1*, and *THRB*). Collectively, our observation of predominant gene hypomethylation in NRAS^Q61^ melanomas and the identification of NRAS^Q61^-linked pathways will be useful for the development of targeted therapies against melanomas harboring NRAS^Q61^ mutations.

## INTRODUCTION

Melanoma is a malignant skin tumor that originates from melanocytes and accounts for more than 70% of skin cancer deaths. In contrast to the stable or declining rates for most other cancer types, melanoma is on the rise with 76,100 new cases and 9,710 deaths estimated for 2014 in the United States [1]. To date, many driver mutations in genes that encode signaling proteins critical for cellular proliferation and survival have been identified in melanomas. BRAF mutations, primarily at codon V600, are the most prominent oncogenic event in melanoma, present in 40-50% of melanomas [2-4]. NRAS mutations, primarily at codon Q61, are the second most common melanoma driver event, occurring in 13-25% of melanomas [3, 5, 6]. Understanding which downstream pathways are regulated by specific driver mutations is essential to develop effective targeted therapies for melanoma.

It is well-known that the BRAF^V600E^ mutation plays a fundamental role in the tumorigenesis of melanoma by activating the Ras/Raf/MEK/ERK (MAPK) signaling pathway. Inhibiting the MAPK pathway in BRAF^V600^-mutant melanoma patients with BRAF inhibitors (vemurafenib and dabrafenib) or a MEK inhibitor (trametinib) has improved progression-free and/or overall survival compared to conventional chemotherapy [7-10]. By investigating genome-wide alterations of DNA methylation and gene expression between BRAF-mutated samples and BRAF wild-type samples, it has been shown that BRAF^V600^ regulates other critical pathways and processes [2, 11-15]. For example, using a methylated CpG island amplification/CpG island microarray system, Hou *et al*. found that BRAF^V600E^ signaling induced widespread alterations of gene methylation in melanoma cells [11]. Additionally, Flockhart *et al*. observed differential expression of protein-coding transcripts and long non-coding RNAs (lncRNAs) between matched normal human melanocytes with and without BRAF^V600E^ based on RNA-sequencing, in which BRAF-regulated lncRNA 1 (*BANCR*) was recurrently over-expressed and played a potential functional role in melanoma cell migration [2].

NRAS mutations occur frequently and are almost mutually exclusive of BRAF mutations [4], making it an attractive therapeutic target for patients without BRAF mutations. Most research and drug development targeting NRAS has focused on the MAPK and PI3K/AKT signaling pathways. Although the most active MEK inhibitors have shown promising activity [16], none have been FDA-approved for treatment of NRAS-mutant melanoma. The effect of NRAS mutations on global cell biology and gene expression remains poorly characterized. Recently, microarrays have been used to investigate global alterations of gene expression between cell lines with and without NRAS mutations [14, 15]. In addition, Jonsson *et al*. observed increased p16^INK4A^ promoter methylation in NRAS-mutated samples compared to NRAS wild-type samples [17]. Therefore, understanding which pathways are affected by NRAS mutations is important and remains a challenge.

In this study, we comprehensively analyzed multi-omics data in NRAS^Q61^-mutated and pan-negative melanomas to identify differentially methylated (DM) genes, differentially expressed (DE) genes, and DE microRNAs (miRNAs) (Fig. S1). Here, in order to eliminate the potential influence of other driver mutations in genes such as *BRAF* and *KIT*, and to focus on genetic cohorts without approved targeted therapies, we limited our analysis to NRAS^Q61^-mutated melanoma and melanomas that are negative for known driver mutations (“pan-negative”) in BRAF, NRAS, KIT, GNAQ, or GNA11. We found that hypomethylation was dominant in NRAS^Q61^-mutated melanomas. Through an integrated functional analysis of DM genes, DE genes, and DE miRNAs, we identified not only the important signaling pathways known to be related to the NRAS^Q61^ mutations, such as the MAPK signaling pathway, PI3K/AKT pathway, CDK4/6/Rb pathway, but also novel processes, such as axon guidance, calcium signaling, and TGF-beta signaling. Finally, we constructed a curated transcription factor (TF) and miRNA coordinated regulatory network, from which we identified downstream regulatory cascades initiated by three concordantly hypomethylated and up-regulated genes (*PDGFD, ZEB1*, and *THRB*) in NRAS^Q61^-mutated melanomas. To our knowledge, this study is the first to integrate DNA methylation, gene expression, and miRNA expression data to systematically analyze potential NRAS^Q61^-associated pathways. Although sample size is not very large in this study, the observations were based on genomic data from the same sample sets. This study suggests novel pathways that may be amenable to therapeutic targeting of NRAS-mutant tumors.

## RESULTS

**Figure 1:**
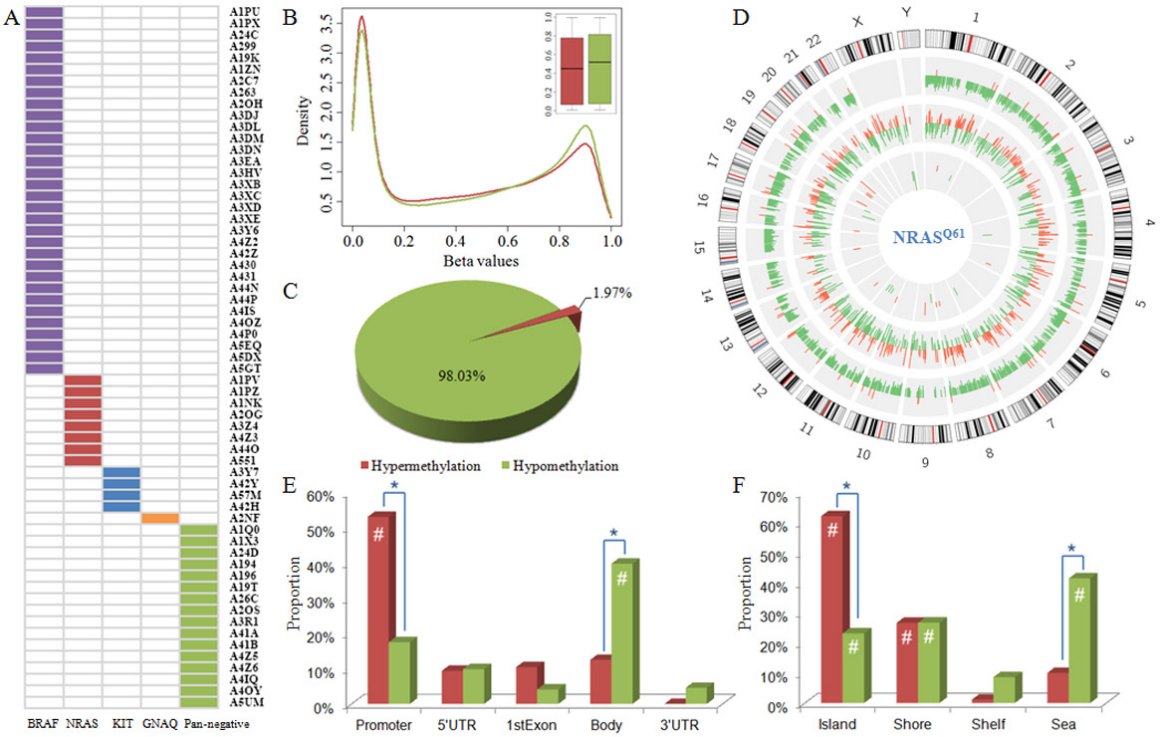
Sample information and global methylation patterns A) Driver mutations in 61 primary melanomas. B) Distribution and box plot of methylation levels in NRAS^Q61^-mutant (in red) and pan-negative (in green) samples. C) Proportions of hypermethylated and hypomethylated probes. D) Circos plot displaying the differences in DNA methylation, gene expression, and miRNA expression in NRAS^Q61^-mutant melanomas compared to pan-negative samples. Each circle from the periphery to the core represents the following: chromosomal location, 4,574 DM CpG sites (hypermethylation in red, hypomethylation in green), 1,150 DE genes (up-regulation in red, down-regulation in green), and 49 DE miRNAs (up-regulation in red, down-regulation in green). The highest bars in DE gene circle and DE miRNA circle indicate the |log_2_FC| is equal to or larger than 5. E) and F) are the distribution of hypermethylated (in red) and hypomethylated (in green) probes across different gene regions and CpG island types, respectively. #, hypergeometric test *p*-value<0.001; *, Fisher's exact test *p*-value<0.001. DM, differentially methylated; DE, differentially expressed.

Genome-wide DNA methylation, gene expression, and miRNA expression profiles for 61 primary melanomas were obtained from The Cancer Genome Atlas (TCGA) (as of February 2014). We defined “pan-negative” samples as those melanomas harboring none of the well-known, specific, and recurrent mutations in the genes *BRAF, NRAS, KIT, GNAQ*, or *GNA11*, which are commonly mutated in melanoma [6, 18, 19]. These driver mutations were mutually exclusive of one another. Of these, eight NRAS^Q61^-mutated and 16 pan-negative samples were identified and used for further analysis (Fig. 1A). We did not observe significant batch effects in DNA methylation (*p*=0.953), gene expression (*p*=0.664), or miRNA expression (*p*=0.715) data (Materials and Methods). The schematic diagram of the proposed integrative genomics approach was shown in Fig. S1.

### Dominant hypomethylation associated with NRAS^Q61^ mutations

Genome-scale DNA methylation levels were examined with the Illumina Infinium HumanMethylation450K BeadChip Kit, which assays for more than 480,000 CpG sites. After excluding probes that were single nucleotide polymorphisms (SNP) associated, located on the X or Y chromosome, or had “NA” values, we obtained 321,892 CpG sites for the following analysis. The relative methylation levels were represented by *β*-values ranging between 0 and 1, measured as the ratio of the methylated probe intensity over the sum of both methylated and unmethylated probe intensities [20]. Comparison of the global methylation (*β*-value) distribution revealed that there was a larger proportion of highly methylated CpG sites in pan-negative samples than in NRAS^Q61^-mutated samples (right peaks in Fig. 1B). Box plots of the *β*-values revealed that pan-negative samples had higher median methylation level than NRAS^Q61^-mutated samples (Fig. 1B).

We next converted *β*-values to *M*-values for statistical analysis (Materials and Methods). *M*-values are represented by the log2 ratio of the intensities of a methylated probe versus an unmethylated probe [20]. The locus-by-locus differential DNA methylation analysis was performed using limma in R package [21] to identify DM CpG sites between NRAS^Q61^ and pan-negative samples. Utilizing the criteria *p*<0.05 and |Δ*M*|>1.5, we determined 90 significantly hypermethylated probes (encompassing 47 genes and 1 miRNA) and 4,484 significantly hypomethylated probes (encompassing 2,085 genes and 28 miRNAs) in NRAS^Q61^ samples (Fig. S2). 98.03% (4484/4574) of DM probes had significantly lower methylation levels in NRAS^Q61^ samples than in pan-negative samples (Fig. 1C and Fig. S3), indicating that NRAS^Q61^ mutations are primarily associated with hypomethylation (Fig. 1D). This result was especially interesting because gene hypomethylation in human cancers is still poorly characterized. Similarly, Hou *et al*. found broad hypomethylation caused by BRAF^V600E^ signaling in melanoma cells [11]. Thus, global hypomethylation induced by or associated with driver mutations appears to be a common feature in melanoma and could potentially play an important role in the pathogenesis of this disease.

Furthermore, we investigated whether the DM CpG sites were significantly enriched (*p*<0.001) in specific functional genomic regions using a hypergeometric test comparing the background distribution of all probes used in this study (Fig. 1E and 1F). In gene regions, we found that hypermethylated probes were significantly enriched in promoter regions, while hypomethylated probes were significantly enriched in gene bodies. In the context of CpG islands, both hypermethylated and hypomethylated probes were enriched in CpG islands and island shores, whereas hypomethylated probes were also enriched in the open sea. These results indicate that the hypermethylated probes were preferentially located in promoter regions and near CpG islands. In contrast, hypomethylated probes were preferentially located in gene bodies and broadly distributed across different CpG island types.

Next, we compared the differences in proportion between hypermethylated and hypomethylated CpG sites in specific functional genomic regions using Fisher's exact test (Fig. 1E and 1F). These results revealed that there was a larger proportion of hypermethylated sites in promoters and CpG islands but a larger proportion of hypomethylated sites in gene bodies and CpG island open sea, which are consistent with previous observations of certain methylation patterns during tumorigenesis [22, 23].

### Alteration of gene and miRNA expression associated with NRAS^Q61^ mutations

Gene and miRNA expression levels were measured by next-generation sequencing analysis. Based on read counts, we used edgeR R package [24] to identify the DE genes and DE miRNAs. At a significance level of *p*<0.05 and log fold change (|log_2_FC|)>1, 1,150 genes were significantly differentially expressed, with 469 up-regulated and 681 down-regulated genes in the NRAS^Q61^ group compared to the pan-negative group (Fig. S4A). Forty-nine miRNAs were significantly differentially expressed, with 26 up-regulated and 23 down-regulated miRNAs in the NRAS^Q61^ group (Fig. S4B). Fig. 1D summarizes the genome-wide alterations of DNA methylation, gene expression, and miRNA expression. It is clear that the hypomethylation associated with NRAS^Q61^ mutations was dominant and widely distributed across all autosomes.

### Signaling pathways linked with NRAS^Q61^ mutations

**Figure 2:**
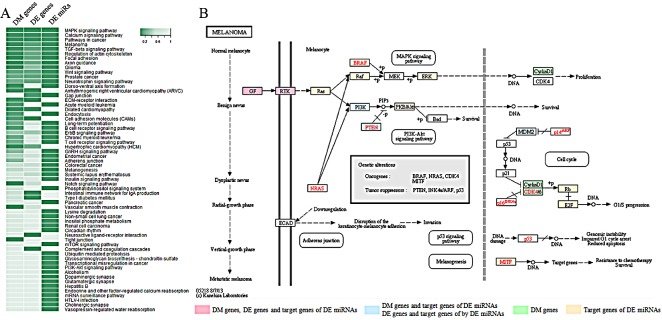
Significant signaling pathways associated with NRAS^Q61^ mutations A) Heat map of enrichment *p*-values for significantly-associated pathways through combination analysis of DM genes, DE genes, and DE miRNAs. B) The “melanoma pathway” in KEGG.

In order to identify pathways associated with NRAS^Q61^ mutation status, we performed an integrated functional analysis of DM genes, DE genes, and DE miRNAs. First, we used the Database for Annotation, Visualization and Integrated Discovery (DAVID) resources [25, 26] to find the pathways that enriched with DM genes or DE genes. Next, we employed the DNA Intelligent Analysis (DIANA) miRPath tool [27] to identify the pathways affected by DE miRNAs. At a significance level of *p*<0.05, we found 16, 10, and 49 significant pathways for DM genes, DE genes, and DE miRNAs, respectively. To incorporate information from multiple levels, we used Fisher's method to combine the three *p*-values for each pathway (Fig. 2A). In the sorted pathway list according to combined *p*-values, we found the most significantly-associated pathway is the MAPK signaling pathway, which is well-known to be dysregulated in most melanomas. This pathway was significant in all the pathway analyses of DM genes (*p*-value: 0.0026), DE genes (*p*-value: 0.0047) and DE miRNAs (*p*-value: 1.11×10^−16^). Current therapeutics are mainly focused on inhibitors of MAPK members, such as BRAF (vemurafenib) and MEK (trametinib) [28]. Both the “Pathways in cancer” and the “Melanoma pathway” were also in the top 5 significant pathways, demonstrating the capability of our approach to capture relevant pathways.

We further investigated the “Melanoma pathway” in KEGG (Kyoto Encyclopedia of Genes and Genomes) (Fig. 2B), and it was clear that the DM genes, DE genes, and target genes of DE miRNAs were enriched in three sub-pathways, which have previously been implicated as downstream targets of NRAS: Ras/Raf/MEK/ERK (MAPK), PI3K/AKT, and CDK4/6/Rb. The PI3K/AKT pathway is another important signaling pathway that participates in both melanoma initiation and therapeutic resistance [29]. For NRAS-mutant tumors, it has been suggested that the combined inhibition of BRAF, MEK or PI3K may be an effective treatment [30]. Multiple novel inhibitors against the PI3K/AKT pathway are currently being studied in clinical trials. In addition, CDK4 is a well-known regulator of the Rb-regulated G1/S cell cycle checkpoint, which is differentially affected by genetic NRAS^Q61K^ extinction [31]. Therefore, targeting the cell cycle via CDK inhibitors in NRAS-mutant melanoma may be a promising strategy [32]. Specifically, the CDK4/6 inhibitor PD-0332991(Palbociclib) has demonstrated anti-tumor activity in melanoma [33]. Combined inhibition of MEK and CDK4/6 is another possible approach and in fact, phase I/II clinical trials have shown promising early results for LEE011 (CDK4/6 inhibitor) and binimetinib (MEK162; MEK inhibitor) [34]. Fig. 2B shows MDM2 as an upstream regulator of CDK4/6, and our analysis suggests it is differentially expressed in NRAS^Q61^-mutant samples. The simultaneous blockade of MEK and MDM2 induces apoptosis in acute myeloid leukemia, indicating the therapeutic potential of this combination [35]. A clinical trial of MEK and MDM2 inhibition in melanoma is planned at Vanderbilt University Medical Center.

In addition to the above known melanoma-associated pathways, we found that the calcium signaling pathway, the TGF-beta signaling pathway, and the Wnt signaling pathway were significantly associated with NRAS-mutant melanoma. Furthermore, we observed that processes involving regulation of the actin cytoskeleton, focal adhesion, and axon guidance, significantly linked with NRAS mutations. Involvement of these pathways is supported by recent findings, and may be novel candidate pathways in melanoma pathogenesis. For example, in melanoma cells and melanocytes, genes down-regulated by MAPK signaling were most often associated with axon guidance, including plexin-semaphorin family members [36], which have been shown to inhibit migration and proliferation in melanoma [37]. These findings could explain why patients with NRAS-mutant melanoma are more likely to have brain metastases [38].

In addition to neuron-related processes, we found B-cell receptor and T-cell receptor signaling pathways in the most highly associated pathways. Various immunotherapy strategies that activate an anti-tumor immune response are playing an expanding role in melanoma therapy. T-cell transfer immunotherapy that uses tumor-infiltrating lymphocytes and high dose interleukin-2 has demonstrated durable, complete responses for many years in patients with metastatic melanoma [39-41]. More recently, immune checkpoint modulators, which activate suppressed cytotoxic T cells have produced long-lasting responses in an increasing proportion of patients [42-44]. Novel approaches also suggest that B cells could similarly be leveraged in melanoma therapies [45]. A differential effect of immune therapy by genotype has been observed clinically; retrospective studies suggest that NRAS-mutant melanomas may respond better to immune therapy compared to other genetic cohorts [46, 47].

### Relationship between DNA methylation and gene expression alterations

**Figure 3:**
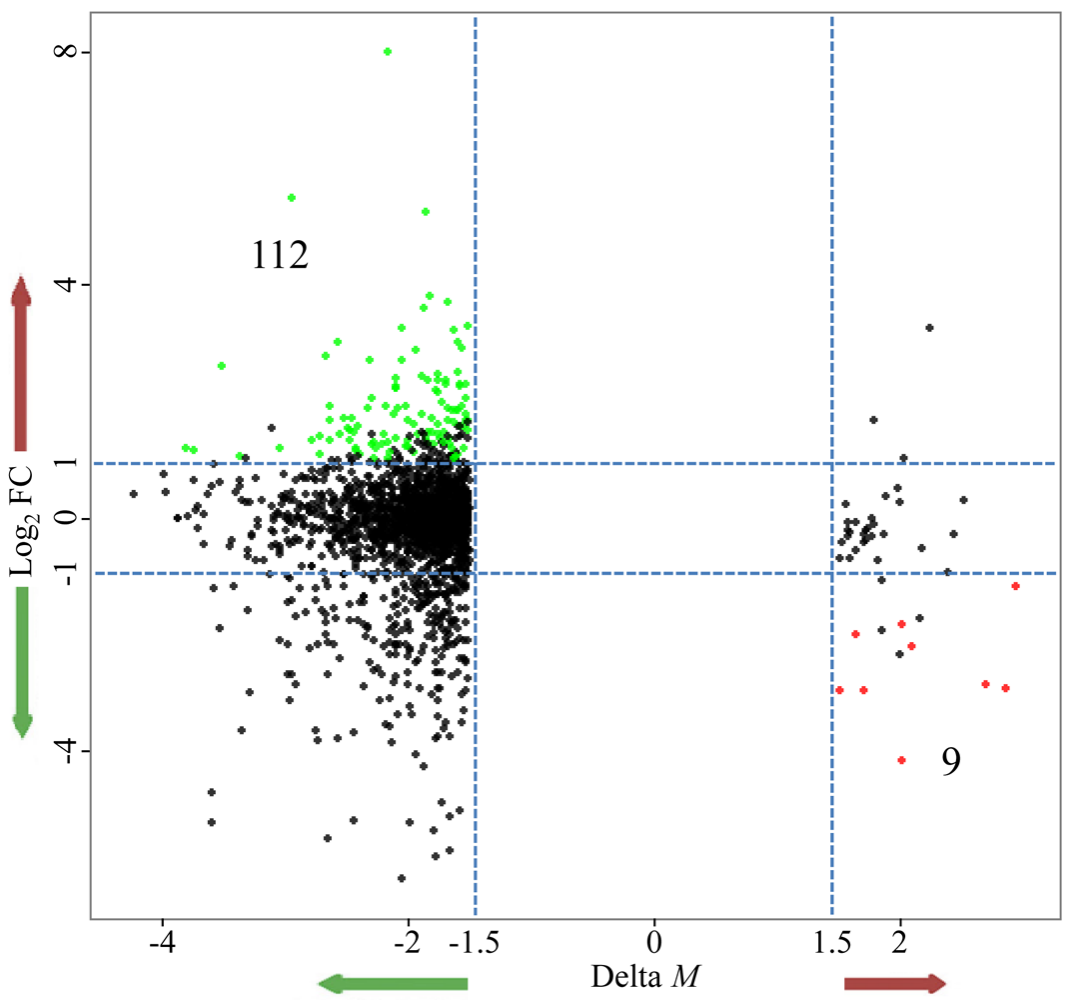
Starburst plot integrating alterations in DNA methylation and gene expression The *x*-axis is the difference in DNA methylation levels (Δ*M*); the *y*-axis is the difference in gene expression (log_2_FC); green nodes represent the hypomethylated/up-regulated genes; red nodes represent the hypermethylated/down-regulated genes.

In order to identify DNA methylation events with potential biological function, we integrated DNA methylation analysis with gene expression analysis. We first calculated the Spearman's rank correlation coefficient between DNA methylation and gene expression for DM CpG sites and their corresponding genes. We found that the hypermethylated loci displayed a stronger inverse relationship with the expression of their corresponding genes than the hypomethylated probes (Fig. S5). The average correlation coefficient was −0.31 for hypermethylated loci and −0.04 for hypomethylated loci. Next, we deeply analyzed the correlation distribution in different gene regions (Fig. S6). For both hypermethylation and hypomethylation, we observed a higher correlation in regions near the TSS (TSS1500, TSS200, 5′UTR, and 1stExon) and a lower correlation in regions far away from the TSS (gene body and 3′UTR). In the above DM loci analysis, we found that the hypermethylated and hypomethylated loci were most commonly observed in promoters and gene bodies, respectively. Thus, these results were consistent with the previous findings that promoter methylation levels negatively correlated with gene expression, while positive correlations were observed between DNA methylation levels in gene bodies and gene expression [48-50]. In the following analysis, we focused on the inverse relationship between DNA methylation and gene expression changes to identify potentially important factors and their regulatory pathways that correlated with NRAS^Q61^ signaling. We obtained 112 genes that were concordantly hypomethylated and up-regulated in NRAS^Q61^-mutant samples, and 9 genes that were concordantly hypermethylated and down-regulated in this sample group (Fig. 3).

### Regulatory networks associated with NRAS^Q61^ mutations

**Figure 4:**
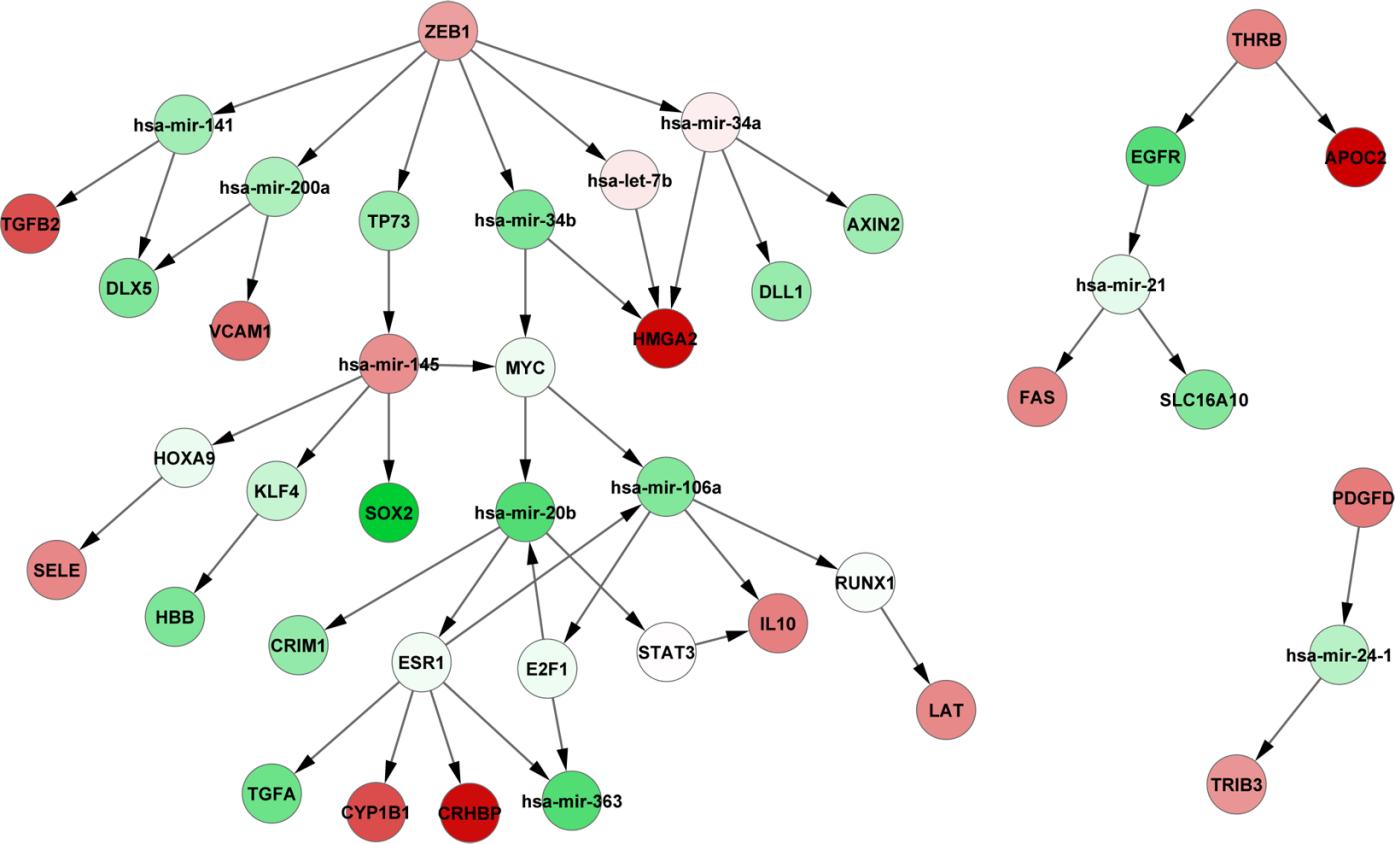
Downstream regulatory cascades of PDGFD, ZEB1, and THRB Green nodes represent down-regulated genes, and red nodes represent up-regulated genes.

We used our previously proposed bioinformatics approach based on Breadth-First-Search algorithm [51] to identify the regulatory cascades that might be affected by aberrant DNA methylation of the 121 above-identified genes (112 hypomethylated/up-regulated and 9 hypermethylated/down-regulated). First, the curated TF and miRNA coordinated regulatory network was constructed through the integration of information from five databases (TRANSFAC [52], TransmiR [53], miRTarBase [54], miRecords [55], and TarBase [56]). This network was used as background network here, which was comprised of TFs, miRNAs and their experimentally validated target genes. 17 of the 121 genes were included in this background network. Next, we mapped all DE genes and DE miRNAs into this network and extracted all DE nodes and their neighbors. In addition, we eliminated the nodes that had outdegree of 0 and were not DE, because we preferred to focus on upstream genes, which might play a critical and causal role. Through these procedures, we constructed a subnetwork associated with NRAS^Q61^ mutations. In this subnetwork, 5 of the 17 genes were included, and three of the genes (*PDGFD, ZEB1*, and *THRB*) had non-zero outdegrees, which indicated that these three genes were regulators of downstream gene expression. All three genes were hypomethylated and up-regulated in NRAS^Q61^-mutant melanoma, indicating that they might contribute to oncogenicity in this subtype. Finally, 52 regulatory cascades originating from PDGFD, ZEB1, and THRB were identified through our previous approach (Fig. 4) [51].

PDGFD is located upstream of the MAPK and PI3K signaling pathways (Fig. 2B) and was differentially methylated, differentially expressed, and targeted by DE miRNAs. PDGFD is one of the members of platelet-derived growth factors (PDGFs), which can regulate many cellular processes in tumors, such as cell proliferation, transformation, invasion, and angiogenesis through specifically binding to and activating its cognate receptor, PDGFR-β [57]. *PDGFD* is highly expressed in the melanoma cell line [58], as our findings had observed. Furthermore, B16 melanoma cells stably transfected with *PDGFD* increased allograft tumor growth compared with cells stably transfected with an empty vector [59].

The aberrant expression of epithelial-mesenchymal transition (EMT) transcription factors, such as ZEB1 (zinc finger E-box binding homeobox 1), can facilitate both neoplastic transformation and tumor cell dissemination [60]. Wels *et al*. found that ZEB1 can promote the migration of melanoma cells through the repression of E-cadherin [61]. Furthermore, Caramel *et al*. demonstrated that *ZEB1* acts as an oncogene and can repress differentiation in malignant melanoma driven by MAPK pathway signaling [60].

Finally, THRB is one of the thyroid hormone receptors (TRs). Many studies have shown that differential expression of *THRB* could be associated with carcinogenesis [62]. Defects in this gene are known to cause generalized thyroid hormone resistance (GTHR) with normal or slightly elevated thyroid stimulating hormone (TSH) levels. The elevation of the circulating levels of TSH is one of the diagnostic hallmarks of hypothyroidism, a condition prevalent in the cutaneous melanoma population [63]. One thyroid hormone, T3, may have an inhibitory effect on melanogenesis in malignant melanocytes [64]. Hypothyroidism can be reversible with use of thyroid hormone replacement [65]. Thus, *THRB*-linked processes and the association between hypothyroidism and NRAS-mutant melanoma should be further experimentally validated, as thyroid hormone therapy might be a novel strategy for NRAS-mutant melanoma treatment.

**Figure 5:**
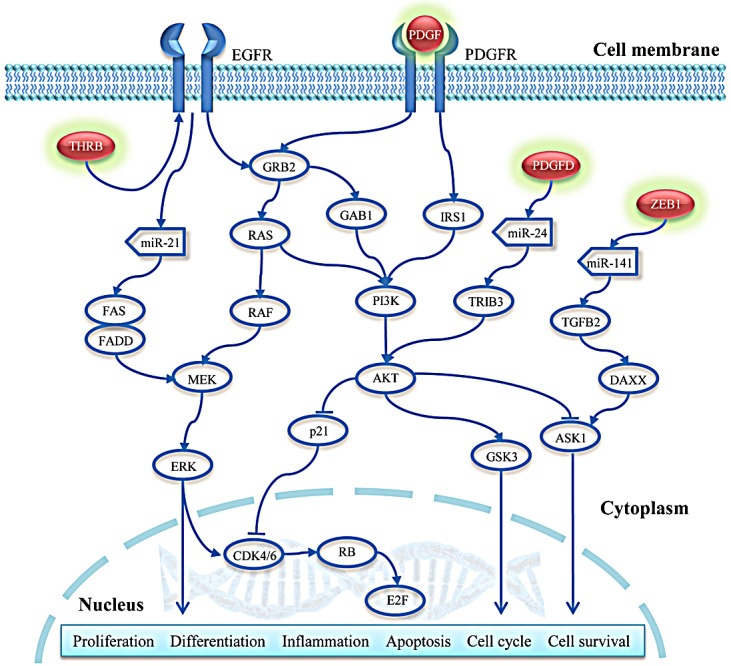
Regulatory pathways potentially associated with NRAS^Q61^ mutations Red nodes with a green shadow represent the hypomethylated and up-regulated genes.

Our results indicate that NRAS^Q61^ mutations are connected to hypomethylation of *PDGFD, ZEB1*, and *THRB*, consequently increasing their gene expression and subsequently dysregulating downstream regulatory cascades. For example, NRAS^Q61^ mutations may induce the hypomethylation of *PDGFD*, which actives its expression (log_2_FC=1.43, *p*-value=0.036). The aberrant expression of *PDGFD* down-regulates the expression of hsa-mir-24-1 (log_2_FC=-0.98, *p*-value=0.042), and then up-regulates the *TRIB3* (log_2_FC=1.15, *p*-value=0.0081). Furthermore, in order to evaluate the therapeutic potentials of *PDGFD, ZEB1*, and *THRB*, we obtained 413 melanoma samples with clinical information and gene expression from TCGA. For each of the three genes, we clustered these samples into two groups according to the average expression level. One included samples with high gene expression (larger than average), whereas another included samples with low gene expression (less than average). The survival analysis revealed that the two sample groups had significantly different survival time (log-rank *p*-values are 0.038, 0.0076, 0.204 for *PDGFD, ZEB1, THRB*, respectively), which indicated that the expression of the three genes were correlated with the patients' survival time. Finally, we sketched the pathways potentially associated with NRAS^Q61^ mutations by integrating the above-identified KEGG pathways with the PDGFD, ZEB1 and THRB regulatory cascades in order to provide a relatively comprehensive landscape (Fig. 5).

## DISCUSSION

Most patients with melanoma harbor BRAF or NRAS mutations. Hitherto, several BRAF inhibitors and a MEK inhibitor have been approved by the FDA and have become standard treatments for those patients with BRAF mutations. Yet, there is still no effective therapy that can block the activity of the mutant NRAS protein. The identification of downstream pathways affected by NRAS mutations is critical to drug development for NRAS-mutant melanomas. In this study, we focused on the predominant NRAS mutations (Q61K/L/R) which makes up over 80% of the oncogenic NRAS mutations in melanoma, and systematically analyzed the potential pathways associated with these NRAS mutations by integrating DNA methylation, gene expression, and miRNA expression data.

In previous studies, researchers identified aberrant DNA methylation and gene expression in samples with driver mutations through comparisons with wild-type samples. In the present study, we compared NRAS^Q61^ mutant melanomas with pan-negative samples, in order to eliminate the effects of other driver mutations and to more clearly assess populations without targeted treatment options. At Vanderbilt, we routinely assess for 43 common somatic mutations in 6 genes in all melanoma patients: *BRAF* (V600), *NRAS* (G12/13, Q61), *KIT* (W557, V559, L576, K642 and D816), *GNAQ* (Q209), *GNA11* (Q209), and *CTNNB1* (S37/S45), using our melanoma SNaPshot assay [4]. Because CTNNB1 mutations usually co-occur with mutations in the other 5 genes, we defined pan-negative samples as those melanomas negative for mutations in the 5 remaining genes [6, 18, 19].

By comparing NRAS^Q61^-mutant samples with pan-negative samples, we identified 4,574 significantly DM CpG sites, 98.03% of which were hypomethylated. This result suggested that NRAS^Q61^-mutated melanomas tend to have lower levels of DNA methylation, and hypomethylated genes may play important roles in the pathogenesis of melanoma. As has been well described, methylation levels and gene expression commonly have an inverse relationship. Thus, decreased methylation levels of oncogenes might explain their over-expression in tumors. Further analysis of the dominant hypomethylation in NRAS^Q61^-mutated melanomas has the potential to identify novel genes with oncogenic properties. Through a functional genomic analysis of DM probes, we found that the hypermethylated and hypomethylated probes were significantly enriched in gene promoter and gene body regions, respectively. The proportion of hypermethylated loci was significantly larger than hypomethylated probes in both promoter regions and in CpG islands, whereas the hypomethelated loci featured prominently in gene bodies and regions far from CpG islands (open sea). We also identified 469 up-regulated genes, 681 down-regulated genes, 26 up-regulated miRNAs, and 23 down-regulated miRNAs in NRAS^Q61^-mutant samples. This result did not reveal any trends regarding differential expression, possibly owing to the complicated positive or negative relationship between gene body methylation and expression levels [66].

In order to identify potential signaling pathways associated with NRAS^Q61^ mutations with high confidence, we used an integrated functional analysis strategy to combine the functional enrichment results of DM genes, DE genes, and DE miRNAs. The MAPK signaling pathway was most significantly associated with this driver subtype, and is well-known to be affected by NRAS mutations. The “melanoma pathway” ranked in the top 5, where we found three sub-pathways that were enriched with DM genes, DE genes, or target genes of DE miRNAs (Ras/Raf/MEK/ERK, PI3K/AKT, and CDK4/6/Rb). The PI3K pathway is another well-known pathway that plays an important role in NRAS-mutant melanomas. The CDK4/6/Rb pathway recently has attracted more attention, and clinically active CDK4/6 inhibitors, such as palbociclib (PD-0332991) and LEE011 are being developed. Two ongoing phase I/II clinical trials are proceeding in NRAS-mutant melanoma combining MEK inhibitors with CDK4/6 inhibitors with promising early results: 1) binimetinib and LEE011 and 2) trametinib and palbociclib. In addition, upstream of the CDK4/6/Rb pathway, we found that MDM2 was differentially expressed, indicating that it might be a potential drug target. In acute myeloid leukemia, a combined MEK/MDM2 blockade may induce apoptosis [35].

We then performed an integrated analysis of DNA methylation and gene expression data to identify DM loci with potential functional significance. We found that hypermethylated probes had a stronger inverse relationship with their corresponding genes than hypomethylated probes. This may be because hypermethylated loci were enriched in gene promoters, which is commonly inversely associated with gene expression, whereas hypomethylated loci were enriched in gene bodies, which could have either a positive or negative gene expression correlation. To identify some key factors that associated with NRAS^Q61^ mutations, we focused on the inverse relationship between alterations of DNA methylation and gene expression. We found 112 genes that were concordantly hypomethylated and up-regulated, and 9 genes that were concordantly hypermethylated and down-regulated in NRAS^Q61^-mutant samples. Our hypothesis was that NRAS^Q61^ mutations induced the aberrant DNA methylation, which in turn affected the expression of key genes; the dysregulated genes then influenced downstream regulation. Starting from the key genes, we employed our previously proposed approach to identify downstream regulatory pathways. After mapping these 121 genes into the curated TF and miRNA regulatory network and extracting the potential subnetwork associated with NRAS^Q61^ mutations, we identified 52 regulatory cascades initiated from PDGFD, ZEB1, and THRB. PDGFD is the upstream regulator of the MAPK and PI3K signaling pathways. The presence of NRAS^Q61^ mutations correlated with a decreased PDGFD methylation level and thus, increased gene expression, which may further induced the activity of the MAPK and PI3K pathways. *ZEB1* is an oncogene that may drive the development of malignant melanoma [60, 61]. Finally, differential expression of one of the thyroid hormone receptors, THRB, may correlate with the high prevalence of hypothyroidism in patients with cutaneous melanoma [63]. One of the thyroid hormones, T3, has been demonstrated to have an inhibitory effect on melanogenesis in malignant melanocytes [64]. Thus, thyroid hormone therapy might be a novel strategy for melanoma drug development. In addition, the expression of *PDGFD, ZEB1*, and *THRB* were also associated with patients' survival time, which indicated that they might have therapeutic potentials.

In summary, we provided novel insights into the downstream pathways that may be associated with NRAS^Q61^ mutations in melanoma. This study was the first to integrate somatic mutations, DNA methylation, gene expression, and miRNA expression to investigate the influence of driver mutations. This analytical strategy can be straightforwardly applied to other mutations in other tumors. One limitation of the present study is a small sample size; however, this is primarily due to our use of strict criteria for the selection of the samples with matched genomic data (methylation, mRNA expression, and miRNA expression), including primary tumors and pan-negative melanomas, in order to eliminate the confounding factors and reduce false positive discoveries. The cumulated multiple levels of genome-wide data will make the analysis more reliable and stable. Our observations could lead to improvements in understanding the effects of NRAS^Q61^ mutations in melanoma and drug development for NRAS-mutant or pan-negative melanomas. In the future, we will use the results from this analysis to experimentally predict promising combination therapies, based on the downstream pathways affected by driver mutations.

## METHODS

### Melanoma samples

Sixty-one primary melanoma samples, which have matched somatic mutation, DNA methylation, gene expression, and miRNA expression data, were selected from skin cutaneous melanoma dataset of TCGA. In order to eliminate the influence of other driver mutations, we selected the samples as controls that were pan-negative for well-known recurrent mutations in five driver genes: *BRAF* (V600), *NRAS* (G12/13, Q61), *KIT* (W557, V559, L576, K642 and D816), *GNAQ* (Q209), and *GNA11* (Q209) [6, 18, 19]. In this study, the somatic mutations were downloaded from the level 2 data of BI mutation calling. We did not find melanomas with GNA11 mutations in the 61 samples, and the other driver mutations were mutually exclusive of one another (Fig. 1A). As a result, there were 8 samples with NRAS^Q61^ mutations and 16 pan-negative samples. In the following analysis, we investigated the alterations of DNA methylation, gene expression, and miRNA expression between NRAS^Q61^-mutated and pan-negative samples.

### Data pre-processing

#### Batch effect

We directly downloaded level 3 data from the JHU-USC HumanMethylation450, UNC IlluminaHiSeq_RNASeqV2, and BCGSC IlluminaHiSeq_miRNASeq for DNA methylation, gene expression, and miRNA expression, respectively. Because all samples came from different batches, we thus used gPCA R package [67] to evaluate the batch effects.

#### Methylation

The DNA methylation data were detected by the Illumina Infinium HumanMethylation450K BeadChip Kit, which interrogates the methylation status of more than 480,000 CpG sites covering 99% of RefSeq genes and 96% of CpG islands. We used the Illumina HumanMethylation450_15017482_v.1.2 manifest file, obtained from http://www.illumina.com, for functional genomic analysis. Here, we removed probes that had SNPs present within <50bp, located on either the X or Y chromosome, or had “NA” values. There were 321,892 CpG sites reserved for further analysis.

#### Expression

For gene and miRNA expression data, we reserved all probes that have non-zero read counts in >16 samples, in order to ensure that there was at least one sample in each sample group. As a result, we obtained 16,506 genes and 427 miRNAs for further analysis.

### Differential methylation analysis

The relative methylation levels were measured as *β*-values ranging from 0 to 1, where values closer to 0 indicated low levels of DNA methylation, and values closer to 1 indicated high levels of DNA methylation. Because *M*-values are statistically valid for differential methylation analysis, we converted the original *β*-values to *M*-values through logistic transformation [20]. Based on the *M*-values, we used limma R package [21] to identify the DM CpG sites between NRAS^Q61^-mutated and pan-negative samples. The limma method uses the linear models and empirical Bayes methods, which can produce stable analyses from experiments with small sample sizes, to assess the difference between the two groups. Because stringent multiple testing may produce a high false-negative rate when the number of samples is small [68], we used *p*<0.05 and *M*-value difference (|Δ*M*|)>1.5 as cutoffs to identify the significant DM CpGs.

For the DM CpG sites, we also performed functional genomic analysis. Utilizing the manifest file from Illumina, the gene regions were divided into six groups: TSS1500 (within 1500 base pairs (bps) of a TSS), TSS200 (within 200 bps of a TSS), 5′UTR (untranslated region), 1stExon (first exon), gene body, and 3′UTR. The CpG sites were grouped in the context of CpG islands: CpG islands, island shores (less than 2 kilobases (kb) from a CpG island), island shelves (2-4 kb from a CpG island), and open sea (more than 4 kb from a CpG island). TSS1500 and TSS200 were considered promoter regions. Here, we used a hypergeometric test to investigate whether or not the hypermethylated or hypomethylated probes were significantly enriched in specific functional regions, and Fisher's exact test to evaluate the difference between proportions of hypermethylated and hypomethylated CpG sites in specific regions. The statistical analysis was performed using R (http://www.r-project.org/).

### Differential expression analysis

Gene and miRNA expression data were generated by next-generation sequencing. Based on read counts, differential expression for genes and miRNAs were assessed using edgeR R package [24]. We defined significantly DE genes or miRNAs if *p*-value threshold <0.05 and |log_2_FC| cutoff >1.

### Integrated functional analysis of DM genes, DE genes, and DE miRNAs

We used DAVID Bioinformatics Resources [25, 26] to examine the enriched KEGG pathways for genes exhibiting altered DNA methylation and gene expression. Next, we investigated the combinatorial effect of the differentially expressed miRNAs on pathways using the DIANA miRPath web server [27]. Here, we used microT-CDS to predict miRNA targets and selected “pathways union” to merge the results. The significance level of all above functional enrichment analysis was set to *p*<0.05. In order to integrate the pathways enriched with DM genes, DE genes, and pathways affected by DE miRNAs, we used Fisher's method to combine the three *p*-values for each pathway. Due to the bias from the extremely small *p*-values, we fixed the *p*-values of all significant pathways as 0.05 in order to incorporate the pathways with moderate *p*-values (such as 0.06 or 0.07). The statistical analysis was performed by R.

### Integrated analysis of DNA methylation and gene expression

For each pair of CpG site and its corresponding gene, we used the Spearman's rank correlation coefficient to measure the correlation between DNA methylation (*β*-value) and gene expression (normalized count). The statistical analysis was performed by R.

### Identification of transcriptional and post-transcriptional regulatory pathways

We first constructed a curated TF and miRNA coordinate regulatory network from five databases, in which all regulations were experimentally validated: TRANSFAC [52], TransmiR [53], miRTarBase [54], miRecords [55], and TarBase [56]. Next, we mapped the DE genes and miRNAs to the regulatory network, extracted them with their neighborhoods, and then eliminated all non-DE nodes with 0-outdegree to construct a subnetwork potentially associated with NRAS^Q61^ mutations. Finally, we used our previously proposed approach based on Breadth-First-Search algorithm [51] to identify all downstream regulatory cascades that started from a specific node.

## SUPPLEMENTARY MATERIAL FIGURES


