# Supplementary material for: Clinically relevant genes and regulatory pathways associated with NRASQ61 mutations in melanoma through an integrative genomics approach
Source: Oncotarget. 2014 Dec 10;6(4):2496–508. doi: 10.18632/oncotarget.2954 (PMC4385866; doi:10.18632/oncotarget.2954)
Supplement: Supplementary file 1 [file oncotarget-06-2496-s001.pdf]

Clinically relevant genes and regulatory pathways associated with NRAS^{Q61} mutations in melanoma through an integrative genomics approach

Supplementary Material

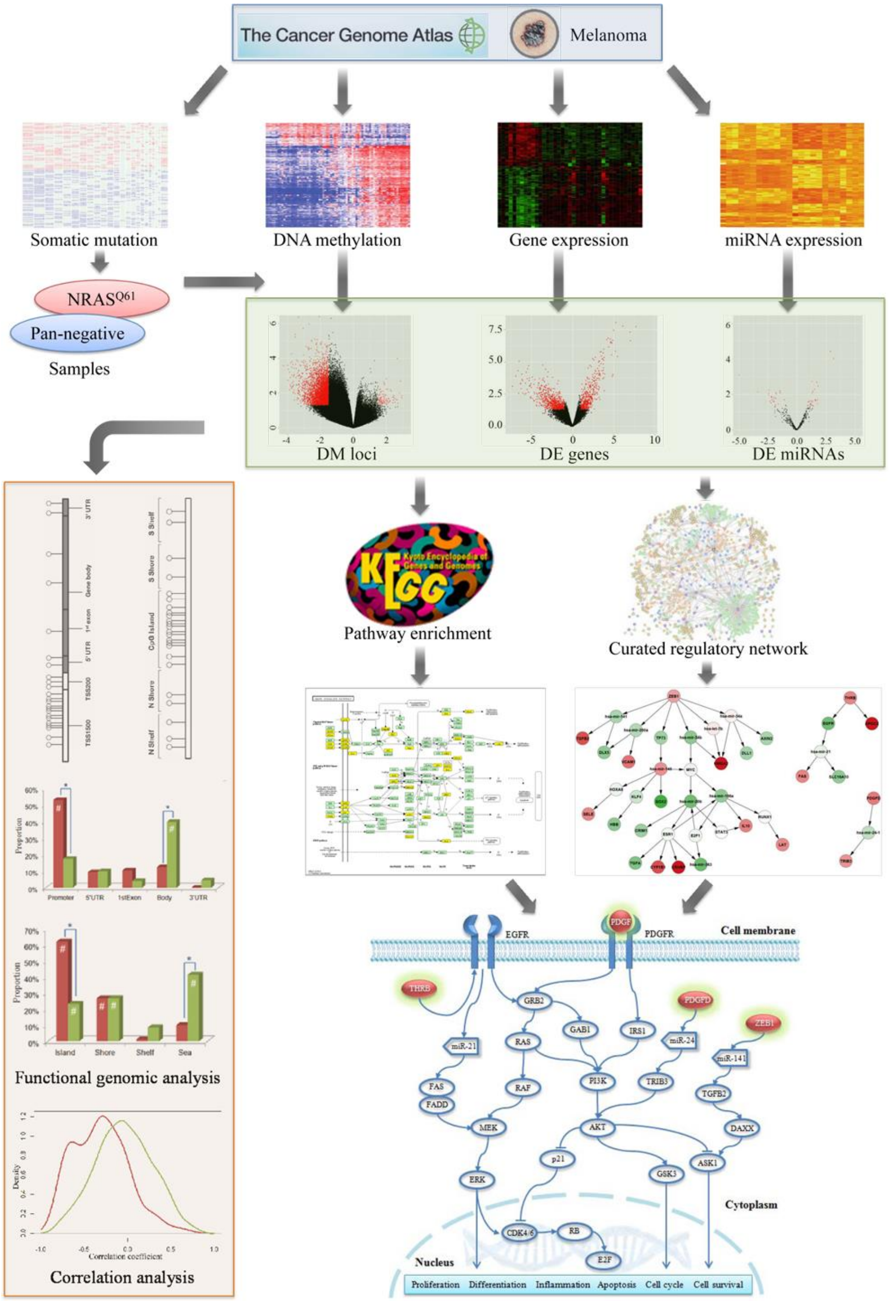

Figure S1: The schematic diagram of this integrative genomics approach.

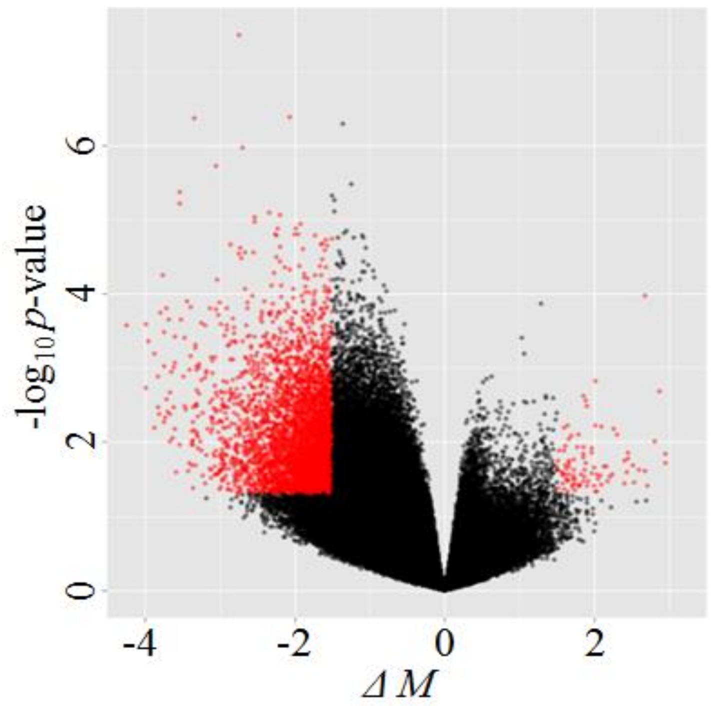

Figure S2: Volcano plot of DM probes between NRAS^{Q61}-mutant and pan-negative samples. x-axis is difference of M value; y-axis is $-\log_{10}p$-value for each probe. Probes with $p<0.05$ and $|\Delta M|>1.5$ were considered significantly differentially methylated (labeled in red).

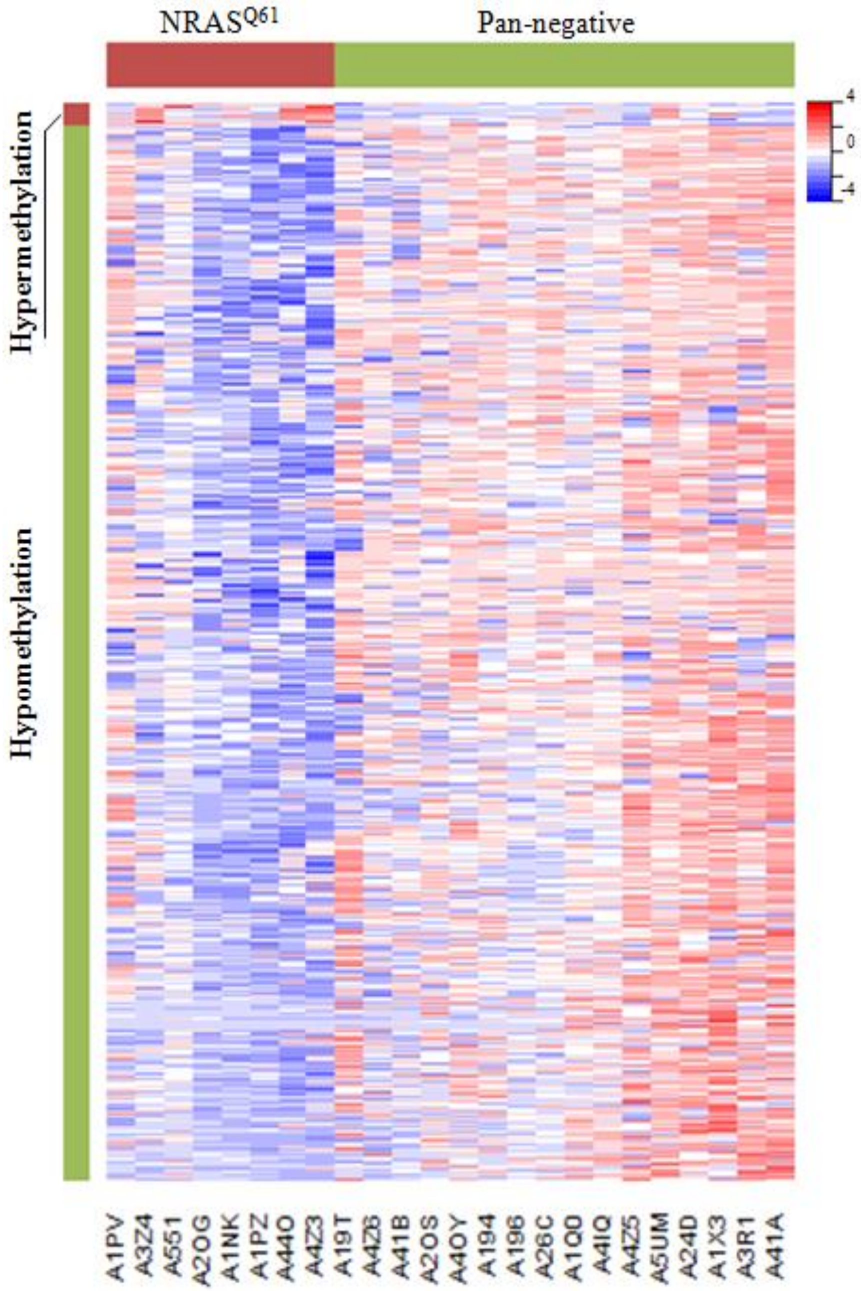

Figure S3: Heat map of the DM probes. The red and green bars above the figure represent NRAS^{Q61}-mutant and pan-negative samples, respectively. The red and green bars to the left side of the figure represent hypermethylated and hypomethylated probes, respectively.

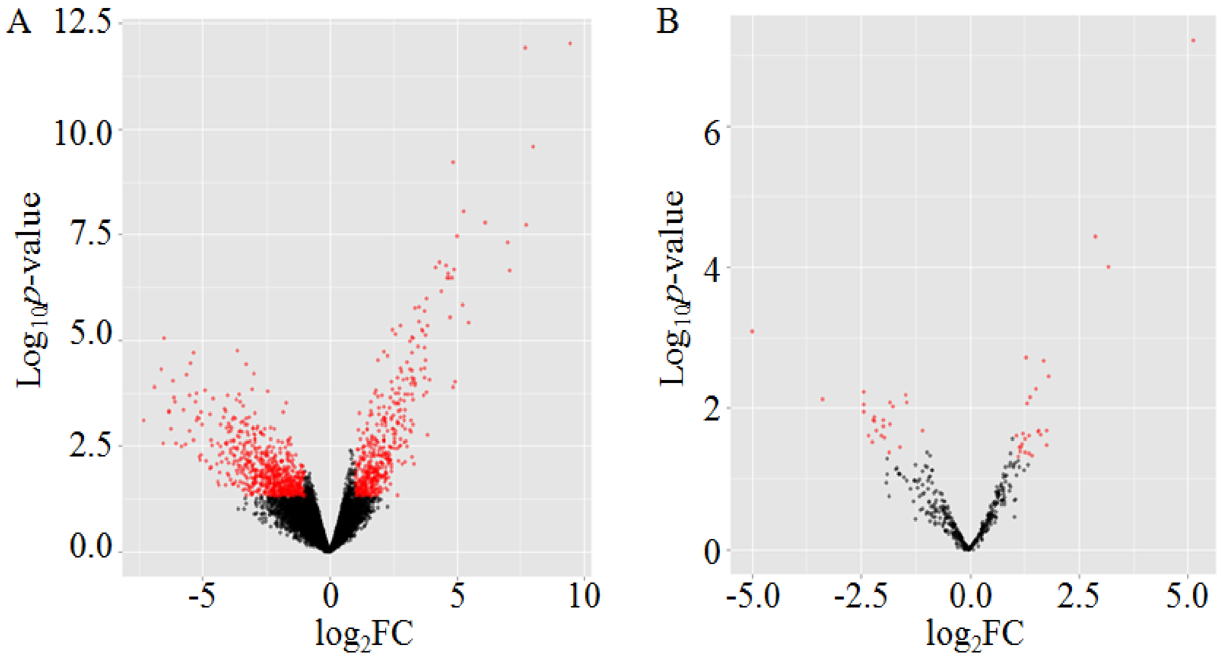

Figure S4: Volcano plots of differentially expressed genes (A) and miRNAs (B). Genes or miRNAs with p<0.05 and $|\log_2FC|>1$ were considered significantly differentially expressed (red).

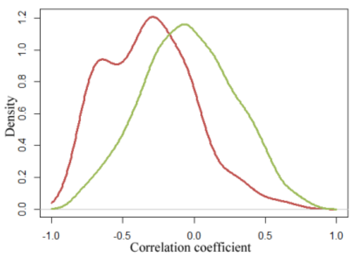

Figure S5: Distribution of correlation coefficients between methylation levels and corresponding gene expression of hypermethylated (red) and hypomethylated (green) CpG sites.

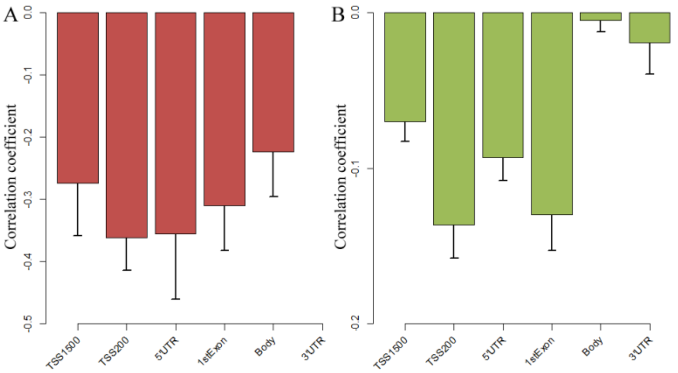

Figure S6: Error bar plots of correlation coefficients between methylation levels and corresponding gene expression of DM CpG sites in different gene regions. A) For hypermethylated probes (in red), and B) For hypomethylated probes (in green).
